# Rationalizing Inconsistent Consumer Behavior. Understanding Pathways That Lead to Negative Spillover of Pro-environmental Behaviors in Daily Life

**DOI:** 10.3389/fpsyg.2021.583596

**Published:** 2021-05-17

**Authors:** Lieke Dreijerink, Michel Handgraaf, Gerrit Antonides

**Affiliations:** Urban Economics Chairgroup, Wageningen University and Research, Wageningen, Netherlands

**Keywords:** negative spillover, rebound effects, moral licensing, justifications, perceived effort

## Abstract

Ideally, pro-environmental consumer behavior leads to a lower impact on the environment. However, due to negative behavioral spillovers environmentally friendly behavior could lead to an overall higher environmental impact if subsequent environmentally unfriendly behavior occurs. In this exploratory interview study we focused on two pathways leading to negative spillover: a psychological path (perceived effort, moral licensing) and an economic path (rebound effects). We wanted to gain insight into people’s motivations to behave environmentally unfriendly and to explore people’s level of awareness of both pathways. Our results indicate that pro-environmental behaviors that are associated with higher effort are performed less frequently, and that when people do not perform these behaviors they associate them with higher effort levels. When people perceive behaviors as more effortful they increasingly seem to use arguments to motivate and rationalize why performing the behavior is difficult or impossible. Moreover, we found that although some people can imagine that moral licensing and rebound effects could occur and can provide examples from their own lives, most people assess these concepts as not rational. People seem unaware of the relation between a first pro-environmental behavior (PEB) and a subsequent behavior, and therefore inconsistencies in behavior go unnoticed. As people are good at rationalizing why they do not perform specific PEBs, they in general feel satisfied with their own pro-environmental actions. In order to discourage negative spillovers, we describe a number of approaches and research ideas aimed at taking away the grounds for rationalization.

## Introduction

Pro-environmental behavior (PEB) takes many forms, such as insulating one’s home, eating less meat, recycling empty glass bottles or using a bicycle. Studies show that people do not behave pro-environmentally consistently. For instance, people can recycle their waste but at the same time make environmentally unfriendly mobility choices (Steg and Vlek, 2009), and saving energy at home does not mean that people save energy while on holidays (Barr et al., 2010). Other studies, however, show that most people do desire to behave consistently (Thøgersen, 2004). Van der Werff and Steg (2018), for example, describe that when people realize they engaged in PEB, their environmental self-identity is likely to be strengthened, thus increasing the likelihood of performing other PEBs. In this case the first behavior leads to the second behavior. This sequence of behaviors is characteristic of behavioral spillover. Originally, Thøgersen (1999) defined spillover in terms of a change in attitude and/or behavior concerning a specific activity produced by a targeted effort at one time that may spill over into related areas at another time. As spillovers can also occur when the first PEB is not caused by a targeted effort (or intervention) and behaviors can also spill over to unrelated areas, we accept a broader definition as a starting point: acting in a pro-environmental way changes a person’s likelihood or extent of performing other PEBs (Lanzini and Thøgersen, 2014). Behavioral spillover can be positive when the adoption of a particular PEB is found to increase a person’s inclination to engage in another PEB. Conversely, spillover can be negative, in which case the reverse effect is observed: after adopting a particular PEB, the probability of an individual adopting another PEB declines (Thøgersen and Crompton, 2009). Studies show that both negative and positive spillovers occur (Galizzi and Whitmarsh, 2019; Maki et al., 2019), but why one or the other occurs remains largely unclear and calls for more research. In this study we focus on the occurrence of negative spillovers. In the literature several examples of negative spillover are described, for instance, people who purchased more green products subsequently consumed greater amounts of water compared to people who purchased less green products (Geng et al., 2016), and people who committed to doing something good for the environment, subsequently donated less to an environmental program (Clot et al., 2016). Spillovers thus imply a correlation between behaviors (e.g., Thøgersen, 2004); only when this correlation is significant the term behavioral spillover is applicable^1^. When there is no correlation between PEBs, behaviors apparently are unrelated activities. If people behave inconsistently with regard to their PEBs, this may therefore be caused either by negative spillover or because behaviors are not perceived as being related. In the latter case people may not experience any inconsistency when performing one behavior and not the other.

Various psychological traits have been identified to explain the occurrence of both types of behavioral spillover, including environmental concern, values (e.g., Van der Werff et al., 2014; Carrico et al., 2018), or preference for consistency (Cialdini et al., 1995). Environmental identity (i.e., the degree to which individuals see themselves as environmentally friendly) in particular has been suggested to play an important role. Sticking to negative spillovers, Truelove et al. (2014) for example suggested that among people with a weak or lacking pro-environmental identity negative spillover may be more likely when behaviors are similar. In addition, Gneezy et al. (2012) showed that the cost of prosocial behaviors serves as a signal of identity and subsequently people behave in line with that self-perception. When initial behaviors are perceived as relatively easy or costless, a person would not perceive him or herself as a prosocial person, and negative spillover is more likely to occur. Truelove et al. (2014) therefore stated that participants’ perceptions of the costs of behaviors are of primary importance to predicting whether or not negative spillover will occur. In line with this recommendation, we investigate the perceived effort of various PEBs in this study. In addition to the role of identity, Gneezy et al. (2012) described licensing as an explanation for the negative spillover observed in their study. A moral license allows people to act without fearing that they will morally discredit themselves (Miller and Effron, 2010). By applying moral licensing people feel free to act immorally after an initial moral act. Negative spillover is often attributed to moral licensing (Galizzi and Whitmarsh, 2019; Maki et al., 2019), which is therefore an important concept in our study of negative spillovers. We will elaborate on this phenomenon in the theoretical framework below.

In economics spillovers at the individual behavioral level also have been studied, albeit using a different terminology^2^. The rebound effect is a well-known example and is particularly relevant to our study as it describes a negative spillover. The rebound effect has been studied widely (e.g., Ehrhardt-Martinez and Laitner, 2010; Ruzzenenti et al., 2019) and concerns how consumers react to a lower price of an energy service by consuming more (for instance lighting) after they took an energy efficiency measure (for instance, buy energy-efficient light bulbs). Because of the lower price, the so-called budget line for a particular energy service shifts and as a result consumer behavior changes: consumers will buy more of the energy service (such as light) than before, as the service has become cheaper. The type of economic decision making as described by the rebound effect only includes economic factors that affect consumption of the particular energy service. However, Santarius et al. (2018) pointed out that over the years the rebound effect has evolved from being considered from a neoclassical economic perspective only, to including several other social scientific disciplines such as psychology and sociology. Nonetheless, research on psychological effects related to rebound effects is still limited. To our knowledge there are no studies into how people perceive rebound effects and if people are aware of the occurrence of rebound effects.

Here, we especially focus on two pathways leading to negative spillovers: a psychological path (perceived effort, moral licensing) and an economic path (rebound effects). Our overall aim is to gain insight into people’s motivation to act environmentally unfriendly. Moreover, we explore people’s level of consciousness of both the psychological and the economic pathway. We define the following research questions: 1. What is the role of perceived effort of PEBs within motivation to act environmentally unfriendly? 2. Are people aware of moral licensing and does it apply to them? 3. Are people aware of rebound effects and do they apply to them? For further exploration, we add a final question: 4. Does perceived environmental impact (such as carbon emission) affect people’s motivation to act environmentally unfriendly? We expect that people use different motivations to explain their environmental decisions, and that effort expended on PEBs plays an important role in these motivations. In addition, we expect that people do apply moral licensing and justification strategies but as these processes are not fully deliberative, we explore how people describe them. Finally, we expect that people are unaware of how they spend the money they save by behaving pro-environmentally. Since we want to investigate people’s opinions, views and use of these concepts in the context of their everyday life, we decided to use a qualitative approach.

## Theoretical Framework

### Perceived Effort of PEBs

The concept of effort is studied across various fields, but proves hard to define. Steele (2020) makes a distinction between actual effort (objective effort), and the perception of that effort (subjective effort). Perceived effort thus builds on actual effort. As we are interested in how people perceive the effort of PEBs and the role of perceived effort within their environmental motivations, in the following we only discuss perceived effort.

A behavior that is perceived as easier to perform is more likely to be adopted, and vice versa: when behaviors are more difficult and require more effort to carry out people are less likely to perform them (Attari et al., 2011; Osbaldiston and Schott, 2011; Urban and Ščasný, 2016). Moreover, among people who are concerned about the environment, the strength of that concern diminishes as behaviors become more difficult or costly (Diekmann and Preisendörfer, 2003). Attari et al. (2011) identified four barriers that affect the ease of behavioral uptake, namely financial, physical, cognitive and temporal barriers. The level of perceived effort of a PEB may therefore be determined by any combination of the perceived effort on these four barriers. For instance, a PEB may be perceived as effortful as one person may associate it with taking up much time and investing physical exertion, while another perceives it as effortful since he or she first has to dive into learning more about it.

**FIGURE 1:**
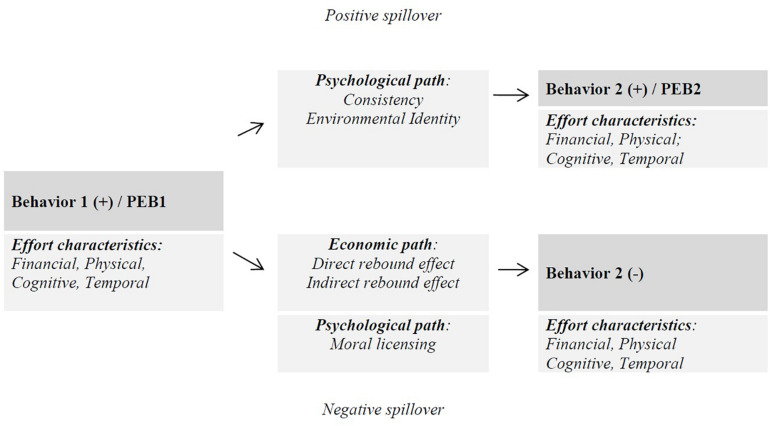
Economic and psychological pathways in positive and negative behavioral spillovers from an initial pro-environmental behavior (PEB1) to a subsequent Behavior 2.

Insight into how people perceive the difficulty of various PEBs is of importance to predicting spillovers (Truelove et al., 2014). There is reason to believe that the sequence of behaviors and their perceived difficulty or effort levels matter for spillover: an easy PEB followed by a difficult PEB may have a different behavioral outcome than the reverse order. The evidence for positive spillover between PEBs of comparable ease, for example, seems strong (Thøgersen and Crompton, 2009), but overall the current state of research paints an incomplete picture. In their meta-analysis Maki et al. (2019) showed that easy first PEBs led to both more positive and more negative spillovers compared to moderately difficult PEBs. Unfortunately, no previous studies tested the effect of a difficult or effortful first PEB on a subsequent PEB, and therefore the meta-analysis could not provide insight into this issue. With regard to prosocial behavior Gneezy et al. (2012) found that costly (in monetary terms) first prosocial behaviors subsequently led to more prosocial behavior, while costless prosocial acts led to less subsequent prosocial behavior. The costs of the subsequent behaviors were not the focal point of the study and were not clearly specified, but we would argue that the subsequent behaviors were costly. In that case their outcome seems in line with findings by Maki et al. (2019) stating that, when the subsequent PEB is difficult, more negative spillovers occur. All in all, in order to explain or predict the occurrence of both negative and positive spillovers, it is necessary to include and consider the perceived difficulty or effort level of the first and subsequent PEBs. Figure 1, describes both the psychological and economic pathways of negative spillover, to be considered next.

### Psychological Pathway: Moral Licensing

As described, by applying moral licensing people feel free to act immorally after an initial moral act. For instance, after investing in an energy-efficiency measure, a person may feel morally permitted to be less frugal with energy. The licensing phenomenon was first introduced in relation to moral issues but has also been studied in the context of consumer behavior, health and eating behavior—under the name of self-licensing. Adriaanse and Prinsen (2017) described that licensing effects are not domain-specific. Behaving morally does not only license subsequent immoral behavior, but unhealthy food choices as well. Consumer behavior studies have found similar cross-domain effects, by demonstrating that respondents were more likely to choose luxury over necessary goods when they just had committed to a charitable act (e.g., Khan and Dhar, 2006). Negative spillover is often attributed to moral licensing (Galizzi and Whitmarsh, 2019; Maki et al., 2019). Additionally, moral licensing is used as a psychological explanation for the rebound effect (Friedrichsmeier and Matthies, 2015).

It is unclear if processes of moral licensing and self-licensing take place unconsciously or consciously. Khan and Dhar (2006) showed that consumers may be unaware of how their prior decisions influence their subsequent choices, and therefore that the process underlying the moral licensing effect may be largely unconscious. Blanken et al. (2015) also state that people may not consciously feel that after displaying certain good behavior “A” they can now engage in undesirable behavior “B.” However, perhaps people who deliberate on a dilemma in which they would like to engage in undesirable behavior “B” (e.g., driving their car for a short distance) are more likely to find a reason why that is acceptable after having just performed a good action (e.g., separating their plastic waste). In that case moral licensing would be a deliberate justification strategy to excuse morally questionable behaviors. This process fits the self-licensing definition of De Witt Huberts et al. (2014, p. 121): “the act of making excuses for one’s discrepant behavior before actual enactment, such that the prospective failure is made acceptable for oneself.” De Witt Huberts et al. (2014) state that self-licensing is not only about being more likely to give in to temptation in response to feelings of deservingness after having behaved responsibly, but also encompasses active engagement in using and searching for available justifications. We therefore hypothesize that people do not take into account prior pro-environmental decisions in a fully deliberate way and we expect that people let these prior decisions influence their subsequent choice. Finding justifications for one’s environmentally unfriendly choices is part of this process. As De Witt Huberts et al. (2014) described, there need to be impulsive motivations that interfere with long-term goals, otherwise justification processes are unnecessary. A justification functions as some kind of credential that then serves as a license to choose an option that would otherwise create negative attributions for the self, such as acting against one’s intentions. Anything can act as a justification and the number of justifications can be infinite, as long as it is generated during a self-regulation dilemma and as long as it forms an allowance that acts against achieving one’s long-term goal. In the present study we aim at exploring licensing and justifications by asking people a number of questions in personal in-depth interviews. This approach implies that we mainly collect information on rational and conscious attributes and not so much on the undeliberate aspects of licensing.

### Economic Pathway: Rebound Effects

The rebound effect is an economic explanation of a negative spillover (see Figure 1). The rebound effect is commonly used in economics as an umbrella term for a number of mechanisms reducing the impact of energy savings achieved from improvements in energy efficiency (Sorrell, 2012). The economic literature identifies three types of rebound effects that encompass both micro- and macroeconomic perspectives: the direct rebound effect, the indirect rebound effect and economy-wide effects (e.g., Greening et al., 2000; Sorrell et al., 2009; Aydin et al., 2017).

The direct rebound effect occurs when an energy efficiency improvement for a particular energy service reduces the price of this service (Verboven and Vanherck, 2016; Aydin et al., 2017). As a result of a combination of the income effect and the substitution effect the consumption of the same energy service increases. The income effect reflects the increase in purchasing power due to a lower price of the service. The substitution effect describes that the lower price of a service may shift consumption patterns to an increased purchase of this service instead of more expensive alternatives. For example, energy-efficient light bulbs make lighting cheaper, thereby encouraging people to illuminate larger areas to higher levels over longer periods of time (Chitnis et al., 2013). Since lighting is cheaper, people can afford to use these light bulbs more often as they have more money to spend (income effect) and this type of lighting is attractive since it is cheaper than other types of lighting (substitution effect). In Figure 1 the direct rebound effect is depicted at the bottom, economic pathway. Using the previous example, the first behavior (PEB1) is a person buying energy-efficient light bulbs, and subsequently this person performs Behavior (2) that has a negative environmental effect: illuminating larger areas to higher levels over longer periods of time. Note that within this sequence of behaviors associated with the rebound effect there is also a PEB0 that is similar to Behavior 2, namely light consumption.

The indirect rebound effect occurs when the reduction of the price of the energy service leads to changes in demand for other goods and services that also require energy or resources (Verboven and Vanherck, 2016; Aydin et al., 2017). In other words, the indirect rebound is about how one spends the money one saves, on other goods or services (Jenkins et al., 2011). For example, cost savings from more energy efficient lighting may be spent on an overseas holiday (Chitnis et al., 2013). The indirect rebound effect can also be explained by income and substitution effects. The economy-wide rebound effect represents the sum of the direct and indirect effects (Sorrell, 2007). In Figure 1 the indirect rebound effect is also depicted at the bottom, economic pathway. In this case the first behavior (PEB1) is, for example, again a person buying energy-efficient light bulbs, and subsequently this person performs Behavior 2 that has a negative environmental effect: saving up for an overseas holiday. Note that in this case there also is a PEB0 (light consumption) but this behavior is dissimilar to Behavior 2.

## Materials and Methods

### Participants

From December 2017 until the beginning of October 2018 we conducted 26 semi-structured face-to-face interviews. Respondents were recruited via family, colleagues, Facebook, and community websites (such as Nextdoor). All interviews were conducted by one researcher, in the Dutch language. We aimed for a mixed group of participants that varied in income level (high, medium, low), gender (male, female) and age (under 30, 30--40, 40--55, over 55), of in total 24 respondents. In practice, when organizing the interviews, some cells were filled easily and more frequently, while others were not. The final group of 26 participants was a good mix: respondents varied in age (*M* = 45.5; *SD* = 18), gender (12 males, 14 females), income level (10 above the Dutch modal income^3^, 10 approximately modal income and six below the modal income) and place of residence (from cities to smaller towns, all in the central part of the Netherlands). Participants that were recruited via public channels were offered a reward for their participation: a gift voucher or a donation to charity (value 30 euro). People who were acquainted with the interviewer through family were not offered a reward (*n* = 6), since people participated as a favor. Interviews took on average about 45 min, with a minimum of 25 min and a maximum of 1 hour and 15 min. The interviews took place at Wageningen University (*n* = 4), at people’s homes (*n* = 15), at their place of work (*n* = 1) or in a cafe (*n* = 6). All interviews were recorded (after the participant’s consent) and anonymously transcribed by a student-assistant. Quotes in this paper were translated as literally as possible from Dutch into English.

### Materials and Procedure

#### Interview

During the interview we asked the respondents to answer 15 questions and to complete one task concerning PEBs and effort (the interview scheme is added as a Supplement). The first set of questions was about gaining insight into motivations related to environmental decision making in daily life. First of all we asked people to provide examples of their environmentally friendly behaviors (question 1) and environmentally unfriendly behaviors (q2), in order to learn what kind of behaviors respondents performed and what behaviors were associated with both concepts. Next, we asked them to assess the overall picture of the examples they provided with respect to environmental friendliness (q3), in order to get insight into how respondents weighed different behaviors and to determine their satisfaction with their own behaviors and choices. In addition, to learn if information about carbon emissions or environmental impact would motivate respondents, we asked if they ever thought about the effect (impact) of their behavior on the environment (q4).

Furthermore, the next set of questions was about rebound effects, as we wanted to know how aware respondents were about the occurrence of this phenomenon and how they would assess it. First, respondents were asked to think of an example in which behaving pro-environmentally led to financial savings (q5). Second, we asked what respondents thought about saving money by acting pro-environmentally and subsequently spending these savings in an environmentally unfriendly way (q6). Third, we asked if people ever thought about how to spend money they saved by behaving pro-environmentally (q7).

After the Effort Scoring Task (see next section) we asked respondents questions about moral licensing, in order to gain insight into how aware respondents were about moral licensing, what their thoughts were on the concept and whether or not they would apply it. We asked respondents whether they agreed or disagreed with each of two statements on moral licensing (q9, q10; e.g., “When I do something pro-environmentally that takes a lot of effort, I feel I can behave less pro-environmentally for a while”).

Finally, we asked respondents whether they agreed or disagreed with three statements on having an environmental effort budget (q11, q12, q13; e.g., “I have the feeling I have a limit or budget for behaving pro-environmentally. Some things take too much effort and therefore I do not do them”). With these questions we wanted to gain insight into respondents’ thoughts about having this kind of budget (not reported here).

#### Effort Scoring Task

Halfway during the interview, we used an Effort Scoring Task (q8) to assess the amount of effort participants associated with 18 PEBs. By means of this task we wanted to learn how much effort respondents associated with each PEB and how this affected their motivation to perform these PEBs. Moreover, we wanted to gain more in-depth insight into how respondents substantiate their effort assessments for each of the PEBs.

In order to accomplish a full view of environmental behavior a broad set of PEBs was needed. We therefore selected 18 behaviors from the General Ecological Behavior (GEB) scale version, as described by Arnold et al. (2017), that were suitable for the Dutch situation. We selected the 18 PEBs to represent six consumption domains: housing, mobility, food, leisure, work, clothing and goods. Moreover, we selected PEBs to represent different levels of environmental impact, including low impact behaviors that would lead to avoiding small amounts of greenhouse gas emissions (such as reading or recycling) and high impact behaviors that would lead to avoiding much larger amounts of emissions (for instance not going on holiday by airplane or insulating one’s home). Participants indicated on a 10-point scale whether they thought a PEB would cost them very little (score 1) to very much effort (score 10). They were handed an A3 sheet with the 10-point scale printed on it and 18 pieces of paper with the names of the PEBs. In addition to scoring the PEBs participants were asked to explain the scores they attributed to each of the PEBs.

### Analysis

We analyzed the transcriptions using Atlas.ti, Qualitative Data analysis software, version 8. In total 121 codes were used to code the transcriptions. Codes were defined at the start of the coding process, since we knew specific topics would certainly be discussed (e.g., indirect rebound, or effort). We also added codes to the list during the coding process, often comprising more detailed topics (e.g., compensation, guilty feelings or footprint). We used both content analysis (What concepts are mentioned?) and domain analysis (Who says what?). Since Atlas.ti provided reports for each code, an overview could be obtained directly. Moreover, we looked at co-occurrence of different labels: for example, if people who indicated driving a car also went on holiday by airplane. The co-occurrence tool and the network options in Atlas.ti were used for this purpose.

In addition, we developed an SPSS dataset in which we quantified the main variables: effort per PEB, overall effort score per person, licensing (awareness and occurrence), and the rebound effect (awareness and occurrence). For each respondent we related the effort scores per PEB to whether or not they performed the PEB, resulting in an effort score per respondent for behaviors they did perform and for behaviors they did not perform. We calculated an overall effort balance by subtracting the effort for PEBs they did not perform from the PEBs they did perform.

Following the definition of effort by Attari et al. (2011) we labeled the arguments respondents used to explain the effort score for each PEB being financial, physical, cognitive and/or temporal. In other words, if a respondent described a PEB as being effortful because it took a lot of time, physical exertion and was expensive, we coded their response with the labels temporal, physical and financial, respectively. Next to the four types of effort we added three labels for behaviors being habitual (always do things a certain way) or being affected by the physical surroundings or social context. These three types of arguments were defined during the coding process as the four effort types appeared insufficient to cover all arguments. We analyzed if respondents who did perform or did not perform the PEB used different arguments, and if respondents with different effort balances used different arguments.

### Net Environmental Impact (NEI)

At the start of the interview, we asked respondents to provide examples of their environmental behaviors. For each of these behaviors we made estimations of the average amount of CO_2_ emission in kilograms per year, using internet sources. We relied mainly on the Dutch website of Milieu Centraal (2019) that provides thorough information for the public on environmental impacts based on lifecycle assessments (LCA). For the PEBs we estimated the avoided CO_2_ emissions, while for the environmentally unfriendly behaviors we estimated the realized CO_2_ emission. Since behaviors were not specified with respect to frequency or duration, we were unable to calculate the actual impacts, so we categorized them roughly into low, medium and large effect behaviors. We then made an assessment per respondent of whether the Net Environmental Impact (NEI) (or the sum of their examples) was negative, neutral or positive. We asked two researchers with expertise in calculating environmental impacts (including LCA) to assess our estimations of the NEI of each respondent’s examples. We incorporated their comments into the final NEI estimates.

## Results

### Effort to Behave Pro-environmentally (Effort Scoring Task)

We analyzed the results of the Effort Scoring Task in multiple ways. First, we investigated the effort respondents associated with each PEB and discriminated between whether respondents actually performed the PEB or not. In addition, we looked at the arguments respondents used to explain the effort scores. Furthermore, we analyzed how effort scores differed between respondents by means of calculating an effort balance, and in addition investigated the arguments respondents used to explain the effort scores.

#### Effort of Performing the PEBs

Of the 18 PEBs, cleaning up after a picnic (*M* = 1.1, *SD* = 0.3) and bringing empty glass bottles to the bottle bank (*M* = 1.3, *SD* = 0.5) were assessed as least effortful overall (see Table 1). For all 26 respondents this behavior took no effort, and all respondents stated they actually performed these two behaviors. In the selection of the PEBs for this study, these two behaviors were labeled as low impact behaviors (leading to avoiding small amounts of greenhouse gas emissions).

**TABLE 1:** Mean effort scores and standard deviations for the 18 PEBs with a subdivision of scores when respondents did and did not perform the PEB.

**PEB**	**Total effort score**		**Effort score when performing the PEB**			**Effort score when not performing the PEB**		
	***M***	***SD***	***M***	***SD***	***N***	***M***	***SD***	***N***
Clean up after a picnic	1.1	0.3	1.1	0.3	26			0
Bring empty glass bottles to the bottle bank	1.3	0.5	1.3	0.5	26			0
Do not have towels changed daily when staying in a hotel	2.1	2.0	1.5	0.6	22	8.0	2.8	2^*^
Use public transport or my bike	2.7	2.1	1.8	1.2	21	6.3	1.5	5
Turn off computer screen at work/school when leaving for 10 min	2.8	2.1	1.7	1.1	14	4.7	2.1	9^*^
Wear a sweater at home when it’s cold	2.8	2.7	1.7	1.0	22	8.5	1.0	4
Behave pro-environmental at work/school	3.2	2.0	2.5	1.5	17	4.6	2.1	4^*^
Use public transport or my bike to get to work/school	3.3	2.9	1.8	1.2	17	8.2	1.0	6^*^
Buy seasonal fruits and vegetables	3.6	2.0	2.5	1.6	15	5.1	1.7	11
Read about environmental issues	3.7	2.7	2.9	2.1	20	6.5	2.5	6
Insulate home to keep it warm	4.0	3.0	3.2	2.8	14	5.1	3.1	9
Install solar panels on roof	4.8	3.4	1.3	0.6	3	5.3	3.3	19^*^
Carpool to work/school	4.8	3.4			0	4.8	3.4	18^*^
Repair goods or clothes that break	5.0	2.8	3.2	1.5	15	7.6	2.0	11
Avoid to buy new goods	5.2	2.9	2.2	1.0	10	7.2	1.7	15^*^
Not go on holiday by airplane	5.8	2.9	3.3	2.3	11	7.1	2.4	15
Be a vegetarian	6.4	2.8	1.5	1.0	4	7.3	2.0	22
Do not buy products from un-ecological companies	6.5	2.1	3.5	1.9	4	7.1	1.5	22
Total	3.8	2.3	2.2	1.3	261	6.5	2.1	178

^*^When the sum of Ns did not add up to 26 this was due to respondents stating that the performance of a PEB was not applicable to them.

On the other end, being vegetarian (*M* = 6.4, *SD* = 2.8) and not buying from non-ecological companies (*M* = 6.5, *SD* = 2.1) were associated with the most effort. Being vegetarian was associated with little effort by the four respondents who were vegetarian (*M* = 1.5, *SD* = 1.0). Eating this way was habitual for them. Some indicated there was some effort related to the social context, for example making sure there would be a vegetarian or vegan option when having dinner with friends at their place or in a restaurant. Being vegetarian was, however, associated with high effort by the respondents who were not vegetarian (*M* = 7.3, *SD* = 2.0). They enjoyed and valued the taste of meat and fish and would miss it. Taking the time to learn new recipes and buying other products were put forward as additional reasons why this behavior would be effortful for them. Not buying from non-ecological companies was associated with the highest level of effort, also among the respondents performing the PEB (*M* = 3.5, *SD* = 1.9)—although for this latter group all effort scores are low. They mentioned the time needed to read about companies and products and about visiting specific shops. With regard to greenhouse gas emission the latter two PEBs are effective in avoiding emissions (but others have an even larger impact).

The results show that respondents reported the performance of the 18 PEBs more often (total of 261, *M* = 10.0 per person) than non-performance (total of 178, *M* = 6.8 per person). The PEBs that are generally perceived as less effortful are performed by many, while the PEBs that are perceived as more effortful are performed by fewer respondents. Moreover, respondents associated the PEBs they actually performed with a lower level of effort (*M* = 2.2, *SD* = 1.3) and the PEBs they did not perform with a higher level of effort (*M* = 6.5, *SD* = 2.1).

Next, we analyzed the arguments respondents provided of why the PEBs were effortful. We categorized the arguments about why the behaviors were effortful into financial, physical exertion, cognitive, temporal, habitual, or being affected by the physical surroundings or social context. Respondents who did not perform the PEBs provided more arguments concerning why PEBs were effortful (total of 208) than those who performed the PEBs, see Table 2. Dividing the number of arguments by the total numbers of PEBs that were either performed (261) or not (178), showed that when respondents performed the PEBs they on average named 0.23 arguments and for PEBs they did not perform on average 1.17 arguments were named. Cognitive effort and limitations in the physical surroundings were most frequently used as arguments. Financial arguments were also used, but least often. Respondents who did not perform the PEBs seemed to use the argument of being used to a different behavior (habits) as a reason not to perform the PEB more often, for instance: “We always do our groceries at the local supermarket and never look at environmental aspects” (R19) and “I am just not used to using public transport or my bike. […] A car is more convenient.” (R17). Although the number of respondents to our study was limited, the total number of arguments respondents provided on why the PEBs were effortful was quite large (269). We therefore explored if there might be differences in the type of arguments respondents used when they either performed a PEB or not. By means of a chi-square test we found no difference in the distribution of the number of arguments between the two groups, *X*^2^(6, *N* = 269) = 5.718, *p* = 0.456. The type of effort arguments did not seem to differ between respondents who did or did not perform the PEBs, although the results should be considered with some caution.

**TABLE 2:** Sum of type of explanations of why the PEBs were effortful, subdivided by respondents performing or not performing the PEBs.

**Explanation type**	**Performing the PEBs**		**Not performing the PEBs**		**Total (*n* = 26)**	
	**Count**	**%**	**Count**	**%**	**Count**	**%**
Financial	2	3	11	5	13	5
Physical exertion	10	16	32	15	42	16
Cognitive	18	30	54	26	72	27
Temporal	7	12	19	9	26	10
Habitual	2	3	28	14	30	11
Physical surroundings	17	28	49	24	66	25
Social context	5	8	15	7	20	7
Total	61	100	208	100	269	100

#### Effort Balance

Participants varied with regard to their effort balance (i.e., the effort associated with PEBs they did perform minus the effort associated with PEBs they did not perform) with a mean score of -23 (*SD* = 24; see Table 3). For most respondents the effort balance was negative (*n* = 21); only five respondents had a positive balance. The most positive balance was 14 (R5) while the most negative balance was -73 (R19). Again, the results show that when respondents did perform the PEBs they associated the PEBs with less effort and when they did not perform the PEBs they associated the PEBs with more effort.

**TABLE 3:** Overall results (mean, SD, minimum value, maximum value) of effort balance, effort score when performing, and effort score when not performing the PEBs.

	**Mean**	**SD**	**Min**	**Max**
Effort balance	−22	24	−73	14
Effort sum score of performed PEBs	21	6	10	36
Effort sum score of not performed PEBs	44	21	11	92

Moreover, we calculated the correlations between effort balance and argument types. As the number of respondents was limited, we want to emphasize that these results are mainly indicative. It showed that when the effort balance of respondents became more negative they provided more arguments overall [*r*(24) = −0.71, *p* = 0.000]. Furthermore, the more negative the effort balance the more arguments were provided related to physical exertion [*r*(24) = −0.45, *p* = 0.022], habits [*r*(24) = −0.69, *p* = 0.000], and the physical surroundings [*r*(24) = −0.61, *p* = 0.001]. Respondents with a more negative effort balance, for example, mentioned it needed to be nice and comfortable at home without wearing sweater (R17), or being accustomed to eating meat for a lifetime and loving the taste (R19), or enjoying exploring the world and therefore needing to fly by airplane (R6). The correlations with regard to the other types were not significant: financial [*r*(24) = −0.17, *p* = 0.416], cognitive [*r*(24) = 0.21, *p* = 0.315], temporal [*r*(24) = −0.30, *p* = 0.131], social context [*r*(24) = −0.10, *p* = 0.634], but as most types had a negative correlation coefficient, except for cognitive arguments, this implies that these types were also used more often when the effort balance was increasingly negative. Of the four previously identified barriers that affect the ease of behavioral uptake, cognitive effort seemed the odd one out. When combining the three other effort barriers (physical exertion, financial, temporal) and correlating this with the effort balance we again saw a negative relation [*r*(24) = −0.60, *p* = 0.001]. It could be that cognitive arguments were used more frequently by respondents with a more positive effort balance, as these respondents are more concerned with environmental behavior and accordingly think more about it.

### Awareness and Occurrence of Moral Licensing

Furthermore, we asked if respondents felt allowed to act less pro-environmentally after they did one large effortful PEB, or multiple smaller PEBs that cost little effort. These were two separate questions, but respondents responded almost similarly to these questions: They disagreed with both. There were a few exceptions of respondents who agreed (*n* = 4). One student stated that because of her study in consumer science she believed in permitting herself unconsciously: “It could be that it works like this” (R3). Another respondent stated that he did not behave very pro-environmentally and therefore would not know how he would react. One other respondent first indicated that he disagreed, but on second thought he recognized that he sometimes, after 5 days of eating vegetarian, felt allowed making a stir-fry with chicken. Regarding eating meat, another respondent mentioned that he took some extra meat when eating meat after a vegetarian day.

Although most respondents indicated they disagreed with the suggestion that they would apply moral licensing or compensate for good behavior, we did hear some moral licensing examples at different points during the interviews (see Table 4). Strikingly, most examples were about eating vegetarian or vegan. For instance, one woman described that she recalled feeling OK with throwing away some plastic bags in the regular bin because she ate vegetarian. Another respondent felt that buying a new and not very energy-efficient car was OK because she and her partner decided to become vegetarians. Another respondent described going on a daytrip to Germany by car because he did something pro-environmentally just before (what exactly he couldn’t remember). Furthermore, one respondent recalled that he once participated in a study for which he was not allowed to eat dairy products and then started eating more eggs. We would assess all these initial behaviors (PEB1) as effortful, as respondents refrained from eating meat or dairy products while they were used to eating meat or dairy. When we focus on the Behaviors 2, respondents mentioned both easy behaviors (such as recycling the plastic bags after all) and effortful behaviors (for instance switch to a daytrip by another mode of transport). In other words, moral licensing seemed to apply to situations including an effortful PEB1, followed by both easy and difficult subsequent behaviors. The effort balance levels of the respondents who provided licensing examples varied (see Table 4). However, none of these respondents had a positive effort balance and none of the respondents were at the extreme negative end of the balance level.

**TABLE 4:** Examples of moral licensing, in relation to respondents’ effort balance.

**Resp. no**	**PEB 1**	**Behavior 2**	**Effort balance**
R1	Not eating dairy products	Eat more eggs	−42
R10	Being a vegetarian	Buy a less energy efficient car	−12
R15	Eating vegetarian 5 days in row	Eat chicken on day 6	−11
R23	Being a vegetarian	Put plastic bags in regular bin	−21
R24	Eating vegetarian on day 1	Eat some extra meat the next day	−32

Next to the moral licensing examples we heard a number of striking justifications of why respondents made environmentally unfriendly choices. For example, one respondent mentioned that not having children sometimes came up in discussions with her husband about behaving pro-environmentally: for instance, that she felt that they could go on a holiday by airplane because they did not have children. She added that this was a bit of a joke but at the same time it had some truth to it. Two other respondents described that they, because of other things in life not going well (due to health reasons), felt allowed to go on a holiday by airplane or eat meat or out of season fruits [“Because of my diet […] I’m only allowed to eat strawberries, so I need those in the winter too” (R11)]. Additionally, the statement that life is about joy and happiness was used as an argument for not wanting to act too frugal.

In sum, our results showed a number of examples of moral licensing, but these processes seemed not to be deliberate: most respondents denied moral licensing would apply to them.

### Awareness and Occurrence of the Rebound Effect

Next, we asked two questions related to the direct and indirect rebound effect. First, respondents provided examples of their PEBs that had saved them money. These included home curtailment behaviors (turning down the heat, turning off the lights when not in use, shorter showers), in-home investments (solar panels, new boiler), and decision making about doing something or doing something differently (using bicycle instead of car, buying second-hand). However, six respondents mentioned PEBs being more expensive, for instance buying ecological products or meat substitutes instead of cheaper regular products, solar panels being not that profitable, or that traveling by train being more expensive than by car. This would imply that for these respondents a first PEB would not lead to money saving or a possible rebound effect. An additional six respondents described that behaving pro-environmentally could both be more costly and save money, depending on the behavior.

Second, we asked respondents what they thought of the idea of spending money in an environmentally unfriendly way while they saved this money because of acting pro-environmentally. Many respondents thought this reasoning was not rational: when they did something for the environment then they would not want to cancel it out afterward. “Either it doesn’t interest you or you are a bit stupid” (R15). But others thought that when (other) people behave pro-environmentally purely from a financial motivation it would make sense; this did however not apply to themselves. Some respondents (*n* = 7) could imagine that in practice people (including themselves) would act like this, but that they were unaware of it. One respondent for example stated: “Yes I think it is true. Because we do not own a car, we save money monthly; I think this really adds up. And we use our money to go on holidays. I went to Rome in January and to Cuba a couple of weeks ago, and in the fall we will go on holiday by plane again” (R22).

When respondents were reminded of the examples they gave previously, they indicated that this money stayed in their bank account and that they did not really have an idea of how they spent it. “I am not very concerned with balancing my money” (R24), and “Suppose you have a monthly budget and there is some money left, then you divide this proportionally over the total budget. It will not create a new category in my budget” (R26). The comparison with smoking was made a number of times. Respondents mentioned that when they quit smoking the money they saved just “disappeared.” All in all, respondents thought that the topic of spending the money they saved by behaving pro-environmentally was a quite difficult one and in general they had not considered this issue before. Only six respondents indicated they thought of this before.

### Balancing Environmental Behaviors (Interview)

At the start of the interview we asked respondents to list a number of examples of their own PEBs. As a first top-of-mind example they often initially mentioned separating their waste (nine times) and eating vegetarian or eating less meat (five times). Curtailment behaviors regarding water use (seven times), heating or electricity (eleven times) were mentioned frequently, but more often as a second or third example. Also, mobility choices, such as not owning a car (five times), using a bicycle or public transport (six times) were mentioned as second or third examples. Co-occurrence of behaviors was limited: using one’s bike and using public transport were mentioned together by some respondents (mobility domain), as were saving on electricity and saving on heat (in-home domain).

Next, respondents were asked to give examples of their environmentally unfriendly behavior. They especially mentioned using their car as an example of their environmentally unfriendly behavior (fourteen times), often as a first top-of-mind example (11 times). Going on holiday by airplane, not separating waste or eating meat were also mentioned (six times). Respondents were less able to name two or three examples of their environmentally unfriendly behavior; sometimes they could not think of a second or third example. Again, respondents mentioned a range of different behaviors and therefore we did not see strong co-occurrence between behaviors: using one’s car and eating meat, and using one’s car and going on holiday by plane were mentioned together by a small number of respondents (three and two times, respectively). Finally, we investigated co-occurrence between the PEBs and environmentally unfriendly behaviors but we found none. In other words, performing a certain PEB was not related to a specific environmentally unfriendly behavior and vice versa.

When asked about the total picture of all of their environmentally friendly and unfriendly behavior examples, a majority (*n* = 18) seemed satisfied and only some acknowledged that they could do more (*n* = 6). One respondent indicated that she felt bad about her behavior. She was disappointed that she did not behave more pro-environmentally. Furthermore, respondents were asked if they thought about the environmental impact of their behaviors, for example, in terms of carbon footprint or CO_2_ emissions. Five respondents indicated that they did not think about the impact of their behaviors at all. The others stated that they thought about their impact, but they did not use a clear definition. These respondents did not exactly know what the impact of their behaviors was, but they mentioned they had an idea of the order of magnitude. One respondent, for example, called the way he estimated the environmental impact of his behaviors a “reasoned feeling” (R22). For only a small number of people, thinking about the actual environmental impact played a role in their daily or weekly life.

Our estimations of the NEI of the provided examples showed that for 12 respondents the NEI was positive (their pro-environmental examples more than compensated for their environmentally unfriendly examples, in sum reducing their footprint), for nine respondents the NEI was negative (their environmentally unfriendly examples surpassed their pro-environmental actions) and for five respondents the NEI was somewhat neutral (all pro-environmental examples seemed to be neutralized by the environmentally unfriendly examples). These results show that for about half of the respondents the impact of their pro-environmental examples was negated to quite some extent by their environmentally unfriendly examples: many described low impact PEBs (for instance waste separation or using LED lights) on the pro-environmental side, but high impact examples (for instance go on holiday by plane or frequent use of their car) on the negative side. These results indicate that participants do various pro-environmental things (large and small) and at the same time do other things that (partially) negate the positive environmental effects of their pro-environmental actions. As we do not know the sequence of these behaviors, it is not to say whether positive or negative spillovers occurred between behaviors.

## Discussion

In this study we investigated people’s motivations to act environmentally unfriendly. In line with previous studies we found that people prefer performing easy PEBs over effortful ones and that PEBs that are associated with higher effort are performed less. Moreover, people who do not perform the behaviors associate these behaviors with higher effort levels compared to people who do perform the PEBs. Possibly, people overestimate the effort associated with a behavior they do not perform as they do not know this behavior very well. In contrast, people who do perform the behavior may adjust their effort assessment downward because of possible cognitive dissonance between behaving in a certain way and claiming it to be effortful. Another explanation could be that performing specific PEBs may become habitual and therefore is assessed as less effortful. Furthermore, our results indicate that the more effort people associate with PEBs the more arguments they use to substantiate or justify their behavior. Although the described barriers to act may of course be real and legitimate, the large difference in number of arguments people use when not performing a behavior compared to when they do perform the behaviors seems to imply that people actively engage in searching for available justifications, as other studies also describe (De Witt Huberts et al., 2014; Schütte and Gregory-Smith, 2015). The type of arguments why behaviors are effortful are similar when people perform or do not perform the PEBs: cognitive effort and limitations in the physical surroundings were most frequently used. Moral or financial explanations were hardly used. Because of the limited number of respondents in our study these results should, however, be viewed with some caution.

Most people rejected the idea of allowing oneself to act in an environmentally unfriendly way after doing something pro-environmental. But we did find a number of moral licensing examples. These examples were all related to a difficult first PEB, followed by an easy or difficult second behavior. This is not in line with previous findings or ideas that performing a difficult first behavior would lead to more positive spillover as it would trigger a person’s environmental identity (Truelove et al., 2014; Gneezy et al., 2012). Our results, however, indicate that the people providing the licensing examples were not the ones who acted the most or the least pro-environmentally, but the ones in between. It might be that especially people that have room to improve their environmental behavior, but do not associate these behaviors with too much effort, can reflect on their own inconsistencies. For people who act pro-environmentally often and by conscious choice, the use of licensing as a deliberate justification might not fit their perceived environmental identity. As Lanzini and Thøgersen (2014) describe, if a person holds moral environmental norms of some strength, behavioral inconsistency threatens the individual’s self-perception as a morally reliable person. While on the other hand for people who act less pro-environmentally, who hold weaker moral (environmental) norms and associate PEBs with high effort levels, acting inconsistently is less of an issue: they have no need to find a morally based justification.

Furthermore, as we had expected, people seemed unaware of the occurrence of rebound effects. People acknowledge that the money they save by PEB ends up being spent, but are unaware of how they spend and whether a rebound effect would occur. The rebound effect was perceived by many as not rational. People indicated that when they do something for environmental reasons and accordingly saved money, they would not want to spend that money on something with a highly negative environmental impact. When reflecting on their own daily life and expenses, people did not have an idea how they spend saved money: it mostly stays in their bank account and is spent ‘at some point’. This lack of awareness fits current knowledge that individuals regularly are neither fully informed nor act fully rationally in the economic sense (e.g., Thaler, 1980; Friedrichsmeier and Matthies, 2015). Frederick et al. (2009) for example showed that the assumption that consumers consider the opportunity costs of a purchase and therefore actively think about alternatives that this purchase would displace is incorrect. People often fail to do so. This could be similar for rebound effects: that people do not think about all possible ways to spend the money they saved.

Finally, in general, people were satisfied with how they balanced pro-environmental and environmentally unfriendly behaviors, also when the balance seemed negative. Low-impact PEBs like waste separation or recycling came readily to mind for many people, similar to findings by Reynolds et al. (2010) and Roy et al. (2015). People could more easily name examples of PEBs than of environmentally unfriendly behaviors. This is striking since most daily-life behaviors do in fact have a negative environmental impact. By focusing on performing PEBs instead of avoiding environmentally unfriendly behaviors, people seemed to overestimate their environment-friendliness. In addition, we found a large variation of examples of both pro-environmental and environmentally unfriendly behaviors and almost no co-occurrence between behaviors. This might imply that there are no clear combinations or orders of behaviors. When people do not perceive behaviors as in some way related, acting consistently is not an issue. Moreover, people seemed to quite intuitively assess the environmental impact of their behaviors, but there is reason to believe that especially for high impact activities people’s assessments are flawed (Attari et al., 2010). In that case people underestimate the actual negative impact of their behaviors. The overestimation of environment-friendliness, the lack of co-occurrence, the underestimation of the negative impact and the use of different types of justification may explain people’s optimistic view of their own behavior.

### Limitations, Implications, and Future Research

People’s perceptions of the costs of behaviors are important in predicting whether or not negative spillover will occur. Our study provides insight into the associated effort of 18 PEBs. On average we could distinguish between low and high effort behaviors, but people differed widely in the level of effort they associated with the various PEBs. This was related to the performance of the behaviors. Studies into positive and negative behavioral spillover should take into account the perceived effort levels of both PEB1 and PEB2 or Behavior2, and elaborate on the definition of effort. Furthermore, these studies should consider the difference when people already perform a behavior compared to when this behavior is new to them.

Since people prefer easy and simple behaviors, it would be fruitful to see if there are PEBs that are perceived as low or medium effort and are effective in reducing environmental impact. For instance buying seasonal fruits and vegetables, and insulating one’s home to keep it warm are associated with a medium effort level by people who do not perform them and are in fact quite effective. By zooming in on the reasons why behaviors are perceived as difficult, intervention designers could try and make PEBs easier. For example, the cognitive barrier of having to read and learn which fruits and vegetables are seasonal could be reduced by offering the products in a specific part of a shop or market. Taking away the barriers that affect the ease of behavioral uptake (for example, making a behavior less time consuming or providing infrastructure) would additionally reduce the number of available justification options.

This qualitative study was most suitable for our exploratory purpose. At the same time there are disadvantages to using face-to-face interviews. Social desirability could have led to people being less eager to share environmentally unfriendly examples. However, respondents did not seem to hold back and many of them did mention environmentally unfriendly behaviors, such as going on holiday by plane or eating “lots of meat.” Another limitation of interviews is that we mainly collected information on rational and conscious attributes and not so much on less deliberate aspects of moral licensing and the rebound effect, while we knew these concepts are also of a non-deliberate or unconscious nature. Some respondents struggled with these questions and found them difficult to think through and answer. It could be that our questions were not clear or that we were asking about things that are hard to put into words or realize one would do at all. By asking people to focus on these issues, it is also possible that their answers are biased due to the focusing illusion (Schkade and Kahneman, 1998; Kahneman et al., 2006), explaining why they usually are not aware of certain issues, except when explicitly asked to pay attention. Instead of a picking a focus on the rational side of moral licensing and rebound effects, future studies could combine conscious and unconscious factors, for instance, by doing experiments in which awareness or conscious processes are manipulated, with participant interviews afterward.

Moral licensing and justifications influence daily environmental behaviors, but these concepts need more research. A taxonomy of justification or rationalization strategies seems to be missing (Chatzidakis et al., 2006). In addition, it is unclear how often and when people apply it (Sörqvist and Langeborg, 2019), when and why people can resist and when they indulge (De Witt Huberts et al., 2014), and what exactly drives justifications. What most potential explanatory mechanisms have in common is that they seemingly allow a person to cross their own lines while minimizing the psychological harm normally associated with such discrepant behavior (De Witt Huberts et al., 2014). Studies on ethical decision making show a similar mechanism: using minor lies allows people to simultaneously benefit financially while keeping up a self-image of an honest person (Shalvi et al., 2011). Future studies could explore whether these personal boundaries or the definition of one’s self-image could be moved toward more environmentally friendliness and if this is also applicable when people hold weaker environmental norms. Furthermore, previous studies show that when justifications are more available people are more inclined to behave unethically. Reducing the number of justifications, by taking away the barriers that affect the ease of behavioral uptake could be a step forward.

Finally, we noticed that people have different associations with environmental impact: it is about recycling waste and avoiding plastics, but also about biodiversity, the use of chemicals, and buying ecological products. Our carbon impact approach does not necessarily do justice to people’s perceptions of what environmental behavior is. Furthermore, we did not make a full overview of people’s behaviors, and it could be that people did not mention specific behaviors that are either environmentally unfriendly or pro-environmental and we were therefore unable to estimate their total impact. The NEI estimations were a small sidestep we included during our analysis, but to do it more accurately it would be better to ask people more in detail. Furthermore, it would be interesting look into people’s satisfaction with their environmental behavior in relation with their actual and more accurate environmental impact.

## Concluding Remarks

In this exploratory study we wanted to gain insight into people’s motivations to behave environmentally unfriendly and to explore people’s level of awareness of both a psychological and economic pathway leading to negative spillover. Our study shows that people are good at rationalizing why they do not perform specific PEBs. There seems to be no issue, as in general people feel satisfied with their own actions and effort related to acting pro-environmentally. Previous studies describe that people prefer to be consistent. If people would indeed behave consistently pro-environmentally this would substantially add to reducing greenhouse gas emissions. Lanzini and Thøgersen (2014) describe that only for people with strong moral norms the desire to avoid cognitive dissonance creates a drive to behave consistently. For people who have no or only weak moral norms for PEBs it matters little to be inconsistent. We would add that the consistent behavior that most people prefer seems to be more about rationalizing their behavior to keep up their self-image of being a pro-environmental person and less aimed at actual greenhouse gas reduction.

Furthermore, based on our findings we argue that people are unaware of the relation between a first PEB and a subsequent behavior. This also prevents people from realizing that their behavior is inconsistent. Although some people can imagine that moral licensing and rebound effects could occur and can provide examples from their own lives, most people assess these concepts as not rational. We think that for many people this indeed is the case: moral licensing and rebound effects do occur but people are unaware or claim to be unaware. The reporting of both phenomena is consistent with the negative environmental impact and greenhouse gas emissions due to environmentally unfriendly consumer behavior.

Thus, in order to substantially reduce individuals’ environmental impact, focusing on consistency might not be the best approach for a large part of the population. Instead, and in order to discourage negative spillovers, we propose to focus on taking away the grounds for rationalization by, for example, making PEBs easier (e.g., less time consuming or providing better infrastructure), or providing insight into which PEBs are most impactful and effective to undertake.

## Data Availability Statement

The raw data supporting the conclusions of this article will be made available by the authors, without undue reservation.

## Ethics Statement

Ethical review and approval was not required for the study on human participants in accordance with the local legislation and institutional requirements. The participants provided their informed consent to participate in this study.

## Author Contributions

LD performed the interviews, (qualitative and quantitative) analyses, and wrote the first draft of the manuscript. All authors contributed ideas to manuscript revisions, read and discussed the manuscript several times, approved the submissions, and contributed to conception and design of the study.

## Conflict of Interest

The authors declare that the research was conducted in the absence of any commercial or financial relationships that could be construed as a potential conflict of interest.

## Footnotes

1 In addition to behavioral spillover, Nilsson et al. (2017) describe the existence of temporal and contextual spillovers. In this paper we focus on behavioral spillover.

2 In economics the spillover effect is also often used to describe macro effects of events occurring in one nation or region having an effect on the economies of other nations or regions (e.g., Baicker, 2005), which is different from the type of spillover behaviors in our study.

3 The Dutch modal income is the household income before taxes. In 2017 the annual modal income was €36,500 (monthly €2,816).
