# Supplementary material for: Rationalizing Inconsistent Consumer Behavior. Understanding Pathways That Lead to Negative Spillover of Pro-environmental Behaviors in Daily Life
Source: Front Psychol. 2021 May 17;12:583596. doi: 10.3389/fpsyg.2021.583596 (PMC8165382; doi:10.3389/fpsyg.2021.583596)
Supplement: Supplementary file 1 [file Data_Sheet_1.docx]

**SUPPLEMENT 1. Interview Questions**

Q1. Can you give some examples of environmentally-friendly behavior that you do?

Q2. Can you give some examples of environmentally unfriendly behavior that you do?

Q3. How do you view the relationship between your environmentally friendly and environmentally unfriendly behavior?

Q4. Do you ever think about the effect of your behavior on the environment?

Q5. Can you think of an example of your own environmentally-friendly behavior that has saved you money?

Q6. If someone behaves in an environmentally friendly way, for example insulating his house or taking the bicycle instead of the car, he will save energy or fuel and also money. This money can be spent on other things, for instance on things that are not good for the environment. What do you think about this?

Q7. For this interview, have you ever thought about spending money that you save through environmentally friendly behaviour?

Q8. We assume that some forms of environmentally friendly behavior take more effort than others. For the following 18 examples of behavior, can you indicate how much effort it costs you on a scale from 1 (no effort at all) to 10 (very much effort) and explain why.

Table 1. Overview of 18 PEBs used in the Effort Scoring Task

| PEB | Description |
| --- | --- |
| A | Use public transport or my bike to get to work/school |
| B | Turn off computer screen at work/school when leaving for 10 minutes |
| C | Behave pro-environmentally at work/school |
| D | Install solar panels on my roof |
| E | Bring empty glass bottles to the bottle bank |
| F | Carpool to work/school |
| G | Clean up after a picnic |
| H | Buy seasonal fruits and vegetables |
| I | Use public transport or my bike |
| J | Do not have towels changed daily when staying in a hotel |
| K | Be a vegetarian |
| L | Do not buy products from un-ecological companies |
| M | Read about environmental issues |
| N | Not go on holiday by airplane |
| O | Avoid to buy new goods |
| P | Insulate home to keep it warm |
| Q | Wear a sweater at home when it’s cold |
| R | Repair goods or clothes that break |

*Only for those who indicated at Q4 to think about the impact of their behavior on the environment.*

*Q9.* You just indicated that you are thinking about the effect of your behavior on the environment. If you think about the effort you want to put into behaving pro-environmentally, is the environmental impact important?

Can you tell to what extent you agree or disagree with the following four statements:

Q10. If I do something pro-environmental and this costs me a lot of effort, afterwards I behave not pro-environmental for a while.

Q11. If I do a number of smaller, pro-environmental things in succession that, independently of each other, do not take that much effort, afterwards I behave not pro-environmental for a while.

Q12. I feel like I have a limit or budget for the amount of effort I want to put into pro-environmental behavior. At a certain moment I did enough.

Q13. I feel like I have a limit or budget for the amount of effort I want to put into pro-environmental behavior. Some things cost me too much effort and I therefore do not do them.

Q14. Can you imagine that people in general would have a budget or limit for pro-environmental behavior?

Q15. Before this interview, did you ever think about having a budget for pro-environmental behavior?
